# COVID-19 Vaccination-Related Sentiments Analysis: A Case Study Using Worldwide Twitter Dataset

**DOI:** 10.3390/healthcare10030411

**Published:** 2022-02-22

**Authors:** Aijaz Ahmad Reshi, Furqan Rustam, Wajdi Aljedaani, Shabana Shafi, Abdulaziz Alhossan, Ziyad Alrabiah, Ajaz Ahmad, Hessa Alsuwailem, Thamer A. Almangour, Musaad A. Alshammari, Ernesto Lee, Imran Ashraf

**Affiliations:** 1 Department of Computer Science, College of Computer Science and Engineering, Taibah University Al Madinah Al Munawarah, Janadah Bin Umayyah Road, Tayba, Medina 42353, Saudi Arabia; aijazonnet@gmail.com (A.A.R.); bhatshabu@gmail.com (S.S.); 2 Department of Computer Science, Khwaja Fareed University of Engineering and Information Technology, Rahim Yar Khan 64200, Pakistan; furqan.rustam1@gmail.com; 3 Department of Computer Science and Engineering, University of North Texas, Denton, TX 76203, USA; wajdi.j1@gmail.com; 4 Department of Clinical Pharmacy, College of Pharmacy, King Saud University, Riyadh 11451, Saudi Arabia; alhossan@ksu.edu.sa (A.A.); zalrabiah@ksu.edu.sa (Z.A.); aajaz@ksu.edu.sa (A.A.); 439204087@student.ksu.edu.sa (H.A.); talmangour@ksu.edu.sa (T.A.A.); malshammari@ksu.edu.sa (M.A.A.); 5 Department of Computer Science, Broward College, Broward County, FL 33301, USA; 6 Department of Information and Communication Engineering, Yeungnam University, Gyeongsan 38544, Korea

**Keywords:** COVID-19 vaccination, healthcare, sentiment analysis, deep learning, lexicon-based approaches

## Abstract

COVID-19 pandemic has caused a global health crisis, resulting in endless efforts to reduce infections, fatalities, and therapies to mitigate its after-effects. Currently, large and fast-paced vaccination campaigns are in the process to reduce COVID-19 infection and fatality risks. Despite recommendations from governments and medical experts, people show conceptions and perceptions regarding vaccination risks and share their views on social media platforms. Such opinions can be analyzed to determine social trends and devise policies to increase vaccination acceptance. In this regard, this study proposes a methodology for analyzing the global perceptions and perspectives towards COVID-19 vaccination using a worldwide Twitter dataset. The study relies on two techniques to analyze the sentiments: natural language processing and machine learning. To evaluate the performance of the different lexicon-based methods, different machine and deep learning models are studied. In addition, for sentiment classification, the proposed ensemble model named long short-term memory-gated recurrent neural network (LSTM-GRNN) is a combination of LSTM, gated recurrent unit, and recurrent neural networks. Results suggest that the TextBlob shows better results as compared to VADER and AFINN. The proposed LSTM-GRNN shows superior performance with a 95% accuracy and outperforms both machine and deep learning models. Performance analysis with state-of-the-art models proves the significance of the LSTM-GRNN for sentiment analysis.

## 1. Introduction

The coronavirus disease 2019 (COVID-19) pandemic has caused a global health crisis, resulting in endless efforts to reduce infections, fatalities, and therapies to mitigate its after-effects. The World Health Organization (WHO) states that COVID-19 is caused by a virus known as severe acute respiratory syndrome coronavirus-2 (SARS-CoV-2) and its first case was reported in China, in December 2019. Within a few months, the highly contagious disease spread globally and was declared a pandemic in March 2020 by the WHO [1]. The global spread of the disease was primarily caused by a large amount of travel, and secondarily by local contagious connections. For instance, in 2018, an excess of 4 billion people (approximately 6 out of every 10 people in the world) traveled internationally through commercial flights [2]. Given the unprecedented spread of the disease, there have been collaborative global efforts to deal with the pandemic. One of the promising interventions in dealing with the COVID-19 pandemic is the development of a vaccine. A vaccine is defined as a substance that creates adaptive immunity for the body that helps in fighting certain ailments/diseases [3]. The history of vaccines can be traced back to 1796 when Edward Jenner developed the first-ever vaccine against smallpox. There are four categories of vaccines depending on their development: inactivated, live attenuated, conjugate/subunit/polysaccharide, and toxoid (Dai et al., 2019). Vaccines have been developed for various serious and non-serious illnesses, and have greatly helped in disease and death prevention.

With regard to COVID-19, as of February 2021, vaccines such as Sputnik V have shown an efficacy level of 91.6% after several trials involving more than 20,000 participants [4]. It has also been reported that many countries have vaccinated their citizens. Similarly, BioNTech Pfizer has shown 95% accuracy after the second dose and 100% accuracy in children aged 12 to 15 [5,6]. Other vaccines are also being used, such as the AstraZeneca vaccine with an efficacy of 90% [7], Johnson & Johnson with 66% [8], and Moderna with an accuracy of 94.6% [8]. Vaccination has greatly helped in controlling the COVID-19 pandemic in many countries, and vaccinations are continuously administered to further curb the pandemic [9,10].

Despite the clinical trials of the vaccines, even as the people are vaccinated, their acceptance may be affected by various factors across different regions of the world. According to Smith et al. [11], the uptake of vaccines depends on people’s perception of their effects, attitudes towards the vaccine, perceived susceptibility to the disease, social influences, and recommendations about the vaccine, among others. To analyze people’s sentiments and trends regarding the COVID-19 vaccination, this study outlines the following research questions:

- Q1: What are people’s sentiments toward COVID-19 vaccination on the social media platform Twitter?
- Q2: How effective is the proposed approach for tweets’ sentiment classification?

To address the research questions and analyze the global perceptions and perspectives of people towards COVID-19 vaccinations, this study used a worldwide Twitter dataset. The study relies on two techniques to analyze the sentiments and evaluate the performances of the proposed methodology: natural language processing (NLP) and machine learning (ML). Three NLP lexicon-based approaches, such as TextBlob, AFINN, and the valence aware dictionary for sentiment reasoning (VADER), along with three machine learning models, such as random forest (RF), logistic regression (LR), and decision trees (DT) have been used for sentiment analysis. The results would be interesting for different domain experts to understand people’s behavior and devise relevant policies to increase the acceptance of COVID-19 vaccination. Additionally, the outcome of the study concerning time-based sentiment analysis results may lead policymakers to devise effective decisions and better public awareness policies regarding vaccination. The key contributions of this study are as follows.

- This study proposes a methodology to perform a systematic analysis of people’s perceptions and perspectives towards COVID-19 global vaccination. For this purpose, a worldwide dataset has been created by collecting the tweets about people’s sentiments regarding COVID-19 vaccination.
- For determining the polarity of the sentiment into positive, negative, and neutral, TextBlob, VADER, and AFINN lexicon-based approaches were used. Different supervised learning machine learning models were applied to the datasets annotated by these approaches to determine the most accurate model.
- To obtain higher accuracy for sentiment classification, an ensemble model LSTM-GRNN is proposed that comprises long short-term memory, a gated recurrent unit, and neural network. Experimental results are validated by comparing the performance with state-of-the-art approaches.

The rest of the paper is divided into five sections. Section 2 contains the related work. Section 3 describes the proposed approach, machine and deep learning methods, and the dataset used for experiments. Section 4 contains the results and discussion. The conclusion is summarized in Section 5.

## 2. Related Work

To analyze the behavioral patterns and vaccination apprehensions, different studies have been conducted, and different barriers to the success of vaccinations have been identified. Motivational and knowledge transfer means have also been applied for vaccination acceptance [12]. These strategies include mass information campaigns to provide information and an understanding of its importance to the public. The primary concern to devise these awareness campaigns among the masses is to understand people’s perceptions, acceptance levels, and reasons for concern regarding the COVID-19 vaccination. In recent years, social media has become the preferred means to address the public due to its wide use by people around the globe. Researchers and practitioners working in public relations (PR) agree that social media plays a key role in engaging people and framing effective campaigning strategies to reach out to people [13]. For example, the Ref. [4] found that social media has very effective consequences in PR and has a persistent discourse in its literature. The researchers argue that the use of social media in PR may lead to effective and vast engagement, along with a positive impact on public behavior [14].

In the wake of current COVID-19 vaccination efforts, studying the behavioral patterns of people can be very helpful to devise appropriate policies to increase the acceptance of vaccination. The behavioral patterns in the uptake of the COVID-19 vaccine will be greatly influenced by the actors involved in the process, strategies framed by governments, and the concerned authorities. The acquisition of real-time information and making dynamic decisions based on real-time information gathering will greatly affect the success of the vaccination drives. The decision-making process towards an effective and successful vaccination drive may be guided by engagement with the target population by listening and responding to their concerns, expectations, and difficulties related to the vaccination [15]. Several studies have stated that effective policies can be made using the data from social media, such as the study in [16] which performed semantic analysis on the data extracted from Facebook text posts. The proposed framework provides important information from social media platforms to help policy-makers in making governmental policies. The research concludes that the inclusion of semantic information from social media platforms can help devise better policies. In the same way, the study in [17] pointed out that using geo-tagged tweets can be influential to determine the trends of particular cities or countries. Using the crowd-sourced data from social media is helpful to design proper interventions from the governments to control the COVID-19 pandemic. Systematic analysis of social media data can provide the perceptions of society and highlight their needs, especially during a pandemic. Other studies report the use of social media analysis to make public policies by governments. For instance, the study in [18] highlights that the Chinese government used data from ’Sina Weibo’ and other social media platforms to make policies during COVID-19. Although not empathic, government officials used social media data to communicate the desired information about the pandemic to the public. The Refs. [17,19] showed how social media can help governments to make public policies. Sentiment analysis is the area of research concerned with the determination of opinions, thoughts, and feelings of a human population about things, events, institutes, and governments using NLP techniques [20].

For the sentiment analysis, it is not enough to only find and consider individual words in the text. It also requires the analysis of sentences concerning their linguistic constructions. Focusing on words, as well as linguistic properties of the text will deduce the real expression of the sentiment. A linguistic construction analysis is usually done using heuristic methods. Researchers have defined heuristics for sentiment analysis in different domains. For example, the study in [21] performed an analysis on film critics, by assuming the negative scope as the words lying between the initial punctuation mark and the negator. Another study [22] used parts of speech tagging (POS) to generate data for negation scope identification. There are potential challenges involved in sentiment analysis. The process is initiated by the identification and collection of the right content about the topic of interest. The text content analysis is not a simple task, and it poses various challenges due to the natural languages’ vast linguistic subtleties. The sentiment orientation needs to be determined using appropriate classification. These challenges can be handled using various methods and techniques [23]. Sentiment analysis, a branch of computational linguistics, is a classification problem in which text segments are classified as positive, negative, or neutral concerning the topic under study [23].

Authors in the Ref. [24] used different online datasets and proposed preprocessing methods to make the tweet text appropriate for NLP techniques. Naive Bayes and maximum entropy-based classifiers have been used in sentiment analysis. Similarly, the study in [25] explored different methods for sentiment analysis of feedback given by students. These sentiment analyses included support vector machine (SVM), complement Naive Bayes (CNB), Naive Bayes (NB), and maximum entropy. SVM and CNB outperformed in terms of accuracy among these methods. Authors in the Ref. [26] proposed an adaptable approach for sentiment analysis on social media posts. The approach determines the opinion of the targeted population in real-time. The methodology consists of sentiment word building, and tweet classification related to the United States (US) presidential elections conducted in 2016. Authors in the Ref. [27] analyzed the important aspects of social media data and concluded that sentiment analysis can be improved by fusing the social media text with the information related to a social context. A sentiment analysis model, named CRANK, has been proposed based on community partitions for improvements in content classification.

The proposed study uses two techniques for a sentiment analysis lexicon-based NLP approach and ML models for sentiment analysis of worldwide Twitter data containing people’s perceptions and views regarding the COVID-19 vaccination. The sentiment analysis may be used to study the behavioral patterns of Twitter users to understand the concerns of people related to the COVID-19 vaccination.

## 3. Materials and Methods

This study proposed a unified framework to conduct sentiment analysis on a large dataset containing tweets related to the COVID-19 vaccination. The dataset was collected from Twitter using different hashtags. The dataset contains the posted text along with the user ID, their location, and the time of the tweet. Pertaining to the use of different redundant words, unnecessary punctuation, stop words, and special symbols, the dataset was preprocessed to make it clean and suitable for training the machine and deep learning models. Preprocessed data are more suited to the models in obtaining higher classification results. Dataset annotation was carried out using TextBlob, which is a lexicon-based approach. TextBlob annotation saves time as human experts need substantially more time to label the data owing to a large number of tweets. The labeled data were split into training and testing sub-datasets for the selected models. Machine learning and deep learning models were trained using the training data, and their performance was optimized by setting several parameters. Test data were unseen to the models and used to test the models for their performance against accuracy, precision, recall, and the F1 score.

### 3.1. Dataset Description

**Figure 1:**
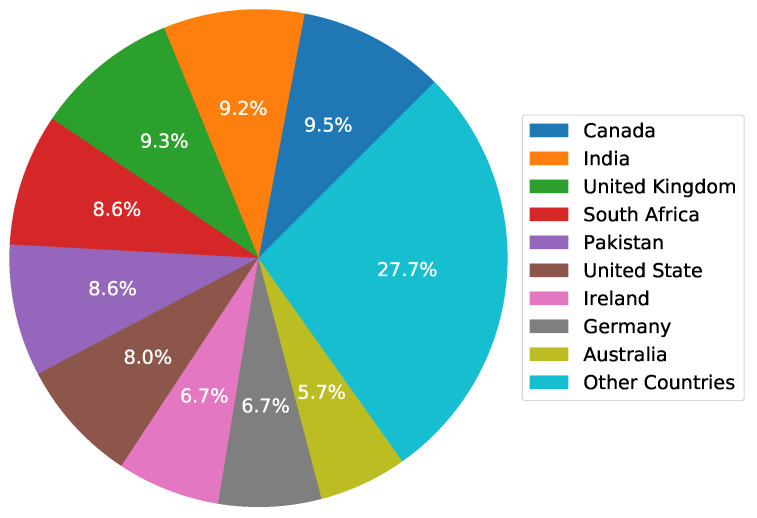
The country-wise number and proportion of tweets.

This study performed sentiment analysis for COVID-19 vaccination. For this purpose, the dataset contains tweets related to the COVID-19 vaccination. To extract the tweets from Twitter, the Tweepy library was used through the Twitter developer account. The tweets were filtered with specific keywords, such as “#Covid19 #Vaccine”, “#Corona #Vaccine”, “#covidvaccine”, “#coronavaccine”, “corona vaccination”, and “covid19 vaccination”, with different geolocations. Various countries have been targeted based on tweet counts related to the topic. The tweets data contained different attributes, such as usernames, locations, text, and so forth. The sample records from the dataset are given in Table 1. The country-wise tweet count and proportion of tweets extracted from each country have been depicted in Figure 1. The collected datasets contain a total of 208 locations that show from where people posted tweets. It is not possible to show all the countries that are tagged with the tweets. Instead, we show only those countries whose number of tweets make at least 2% of the total tweets, i.e., countries with the highest number of tweets are shown in Figure 1. The rest of the countries are labeled ’Other countries’ and include Belgium, New Zealand, Spain, Western Australia, Italy, and many others.

### 3.2. Data Preprocessing

After data extraction, the next step is data preprocessing to remove noise and irrelevant information so that the training process of the selected models can be enhanced.

The cleaning process involves the removal of the data elements in the tweets which are not useful for the sentiment analysis process. Such data elements include @username, # symbols, hyperlinks, punctuation and stop words, and so forth. The preprocessing of tweets was performed using the natural language toolkit (NLTK) library. NLTK incorporates more than 50 corpora, lexical analysis resources, and a collection of libraries for text processing. These text processing libraries contain the important and fundamental NLP functions for tagging, parsing, tokenization, as well as semantic reasoning [28]. The preprocessing steps are explained in subsequent subsections, and some tweet samples before and after preprocessing are shown to show the output of these steps.

#### 3.2.1. Removal of Username, Hashtags, and Hyperlinks

People mostly tag their friends and related persons in their tweets using ‘@username’ on Twitter to refer to or tag them, and also use hashtags and hyperlinks in their tweets. These elements in tweets are not useful for the sentiment analysis, so ‘username’, ‘hashtags’, and ‘hyperlinks’ were removed from the tweets. Table 2 shows the sample text of tweets before and after preprocessing.

#### 3.2.2. Removal of Numbers, Punctuation and Stop Words

Numbers, punctuation marks, and stop words are also not required to find the sentiment in the text. Thus, all non-alphabetic characters, such as numbers and punctuation marks, were removed. Additionally, all the stop words were removed using the NLTK library functions. Table 3 shows the sample text after this preprocessing step.

#### 3.2.3. Case Conversion, Stemming, and Lemmatization

To reduce the complexity of the data and simplify the data, all the resulting tweet text was converted to lowercase letters. The conversion was considered because computers treat the same letter or word in lower case differently than its uppercase form. For instance, ‘a’ is not treated the same as ‘A’, and similarly, ‘GO’, ‘Go’, and ‘go’ are treated as three different words. On the other hand, people in their natural written language use these words for the same meaning. Thus, to bridge this gap between the usability of letters’ case usage among humans and computers, this conversion was done to reduce the complexity. To further simplify the text data for sentiment analysis, two text-normalizing procedures were used, such as stemming and lemmatization.

These text-normalizing techniques were applied to adjust the text to simplify tagging. Since for text in the written human language, a word can have different meanings based on the context in which it is used. For instance, words such as ‘goes’, ‘gone’, and ‘going’ provide an identical meaning considering their root word ‘go’. Thus, concerning a search query or intended information retrieval, the words ‘goes’, ‘going’, or ‘gone’ has no difference in searching for the root word ‘go’. This kind of distinction between the various forms of a single root word is referred to as inflection. The inflection of these words in the tweet texts has been removed to generate the root words from the different inflected words using stemming and lemmatization. These text processing analysis procedures work differently to achieve the desired results. Stemming and lemmatization are applied to change the inflected words to their root word. For instance, all the occurrences of the word ‘goes’, ‘gone’, and ‘going’ were changed to their root word ‘go’. The results after case conversion, stemming, and lemmatization are shown in Table 4, while Table 5 shows the sample text before and after all preprocessing steps have been carried out.

### 3.3. Lexicon-Based Methods

#### 3.3.1. TextBlob

TextBlob is a well-known lexicon-based method for performing various natural language processing (NLP) tasks on the raw text [29]. A Python library, named TextBlob, serves as a programming interface to process text data by using the TextBlob Algorithm 1 implementation. For instance, using the TextBlob, one can analyze sentiments in text, extract noun phrases, create POS tags, translate, classify, and more [30]. In a nutshell, the TextBlob library comes with different in-built functions that assist the task of language processing. It can work for different languages, like Spanish, English, Arabic, and so forth. The TextBlob algorithm for sentiment analysis works in conjunction with NLTK and pattern processing [31]. There are around 2918 lexicons in its dictionary. The polarity calculation is either based on subjectivity (i.e., personal opinions) or objectivity (facts) in TextBlob. The sentiment analyzer returns sentiment scores, such as the (polarity score, subjectivity score) [32].

Table 6 shows the sentiment score range for TextBlob, where scores less than 0 indicate that sentiments have negative polarity, while sentiments with positive polarity have scores above +1.0. As for the subjectivity part, scores below 0.0 indicate that the sentiments are based on facts, while scores above 1.0 show that the sentiments are based on personal opinions.
**Algorithm 1** TextBlob algorithm for sentiment analysis.**Input:** Input: Worldwide COVID-19 Vaccination Tweets**Result:** Polarity Score > 0 ⟶ (Positive)Polarity Score = 0 ⟶ (Neutral)Polarity Score < 0 ⟶ (Negative) initialization **loop** (each tweet in tweets)Compute Polarity Score TextBlob (tweet)**condition:****if** (Polarity Score > 0) **then**Tweet Sentiment = Positive;**elseif** (Polarity Score = 0) **then**Tweet Sentiment = Neutral;**else**Tweet Sentiment = Negative;**condition end****loop end**

#### 3.3.2. Valence Aware Dictionary for Sentiment Reasoning

VADER is a lexicon-based approach that works on gold-standard heuristics with sentiment lexicons written in the English language. The lexicons are scored and validated by humans. They utilize qualitative methods for improving the performance of the sentiment analyzer [33]. Kirli et al. [34] suggests that the scores of the VADER sentiment analyzer hold similar results as that of human raters. The corpus of VADER is a combination of multiple data sets. The previous corpus included only the polarity of the sentiments, whereas VADER has an additional feature that tells the intensity of that polarity score. Its corpus also includes slang words and abbreviations that make more than 7500 lexicons collectively. The range of scores is between −4.0 to +4.0. These values set a threshold for sentiments, such that the scores below −4 indicate the negative sentiments, while the positivity in sentiments is indicated by scores above +4. The output of VADER is something like (neg, neu, pos, compound). Here, the compound score has a range from −1.0 to +1.0 and is based on the aggregated scores of lexicons of a whole text or a sentence. Table 7 shows the sentiment score range for VADER.

The algorithm of VADER (Algorithm 2) involves not just a sentiment lexicon approach, but also the grammatical rules and syntactical conventions for representing the sentiment polarity and intensity. The lexicon approach of VADER contains various lexical features including acronyms and emoticons; therefore, the VADER dictionary contains about 7500 sentiment features. The sentiment intensity of a word is determined through the consideration of grammatical rules and consequently, the sentiment score of a word may vary.
**Algorithm 2** VADER algorithm for sentiment analysis.**Input:** Input: Worldwide Covid19 Vaccination Tweets **Result:** Compound Score>= 0.05 ⟶ (Positive)Compound Score > −0.05 to Compound Score < 0.05 ⟶ (Neutral)Compound Score < 0.05 ⟶ (Negative) initialization **loop** (each tweet in tweets)Compute Compound Score VADER (tweet)**condition:****if** (Compound Score >= 0.05) **then**Tweet Sentiment = Positive;**elseif** (Compound Score > −0.05 to Compound Score < 0.05) **then**Tweet Sentiment = Neutral;**elseif** (Compound Score <= 0.05) **then**Tweet Sentiment = Negative;**condition end****loop end**

#### 3.3.3. AFINN

AFINN is a sentiment lexicon based on the Affective Norms for English Words lexicon (ANEW) in the English language developed by Nielsen, F.A. [35,36]. Similar to VADER, it employs a broad range of words of the English language, with their respective sentiment scores. Unlike VADER, ANEW does not include slang words, and the AFINN lexicon was constructed to bridge this gap. It adopts a rule-based approach, with a manually compiled lexicon. AFINN works in a more general way, is less complicated, and involves fewer computations. The valence scores in AFINN range from −5 to +5, for each lexicon. Positive sentiments have a score above +5, whereas negative sentiments are indicated below −5 [37]. Table 8 shows the sentiment score range for AFINN.

The AFINN lexicon is developed through the observation of the kind of textual data being used on microblogging platforms. Specifically, for Twitter, the people’s posts were collected and regarded as having high sentiments, which led to the increment of the words in the list. The Urban Dictionary was also largely used, which has all kinds of modern acronyms like LOL and ROFL. For given data, it is required to find out the opinion orientation through the list of positive and negative words for every category of data. Therefore, an estimation of the sentiment strength is carried out over the words that carry a sentiment and accordingly, a positive or negative value is assigned to each word (Algorithm 3).

### 3.4. Machine Learning Approaches Used for Experiments

#### 3.4.1. Term Frequency-Inverted Document Frequency Features

TF-IDF is a widely used approach for extracting features. This technique is commonly utilized in music-information correction and text analysis [38]. It allocates weight to the terms in a given document following the inverse frequency of the document and frequency of terms [39,40]. Higher weighted score terms are treated as more important [41]. TF-IDF can be described as

(1) $$tfidf=tf_{t,d}*log\frac{N}{D_{i,t}},$$

where $tf_{\left(t,d\right)}$ is the frequency of term *t* in document *d*, *N* is the number of documents, and $D_{i},t$ is the number of documents containing the term *t*.
**Algorithm 3** AFINN algorithm for sentiment analysis.**Input:** Input: Worldwide Covid19 Vaccination Tweets **Result:** Polarity Score > 0 ⟶ (Positive)Polarity Score = 0 ⟶ (Neutral)Polarity Score < 0 ⟶ (Negative) initialization **loop** (each tweet in tweets)Compute Polarity Score AFINN (tweet)**condition:****if** (Polarity Score > 0) **then**Tweet Sentiment = Positive;**elseif** (Polarity Score = 0) **then**Tweet Sentiment = Neutral;**else**Tweet Sentiment = Negative;**condition end****loop end**

#### 3.4.2. Decision Tree

DT is a machine learning model used in both regressions, as well as classification problems [42]. The model uses the binary approach to split the dataset into an *n* number of subsets continuously, unless the splits become atomic. The atomicity in this context means when a data subset cannot be divided further. Along with splitting the dataset into an incremental approach to building, a decision tree is followed with many branches having a variable size. To reduce the complexity and also overcome model over-fitting, the DT in this study was used with a max_depth hyper-parameter.

#### 3.4.3. Random Forest

Random forest is an ensemble model utilized for constructing predictions with high precision by composing the results of sub-trees. RF employs bagging for training several decision trees by employing samples of bootstrap [43]. The bootstrap samples perform sub-sampling and replace the dataset after training [44]. The RF approach uses decision trees to aid the process of prediction using attribute selection [45]. In the ensemble, the results are merged via voting after the training of models. The most well-known ensemble methods are boosting [46] and bagging [44,47]. Bootstrap aggregation or bagging is an approach in which several models are trained upon bootstrapped samples. An RF can be represented as

(2) $$rf=mode\{tr_{1},tr_{2},tr_{3},\ldots ,tr_{n}\}$$

(3) $$rf=mode\{\sum_{i=1}^{n}tr_{i}\}$$

where $tr_{1},tr_{2},tr_{3},\ldots ,tr_{n}$ are decision trees in RF and *n* is the number of trees.

RF has been applied with up to 300 weak learners for achieving higher accuracy and the *n* estimator value has been set to 300. The n_estimator parameter describes the number of trees added to the prediction process. The parameter max_depth used in the random forest is 60 and has been utilized for setting the maximum depth level. It helps to reduce the probability of the model’s over-fitting [44]. Another parameter, ’random_state’, was used for the randomness of samples at the time of training.

#### 3.4.4. Logistic Regression

LR is another machine learning model widely used for classification, and is based on the concept of probability [48]. LR is a statistical method based on a logistic function. It works with discrete and continuous data, like weight and age. The LR relationship is among the absolute dependent variables and (one or more) independent variables (where the dependent one is usually known as a target class) through calculating probabilities through a logistic function using

(4) $$g\left(x\right)=\frac{L}{1+e^{-k(v-v_{0})}}$$

The values for the variable *v* and S-shaped curve of the logistic function range from −∞ to +∞ for actual numbers. This study utilizes the “liblinear” hyperparameter to boost LR performance as it has a small corpus. The ‘multi-class’ parameter is set to ‘multinomial’ since it is more suitable for binary classification problems. The LR classifier was selected because it is more suitable for binary classification [49].

### 3.5. Deep Learning Models for Sentiment Analysis

To analyze the performance of the deep learning models with regard to the COVID-19 vaccination, this study also leveraged four individual deep learning models. In addition, two ensemble models are proposed. For this purpose, the customized architecture of the convolutional neural network (CNN), long short-term memory (LSTM), recurrent neural networks (RNN), and gated recurrent unit (GRU) is made to obtain higher levels of performance for the task at hand. In addition to individual models, two ensemble models are proposed that comprise CNN-LSTM (ensemble of two models) and LSTM-GRNN (ensemble of three models). These models are deployed using the Tensorflow framework, and the used architecture of these models are shown in Table 9. These models were compiled using the categorical_cross-entropy loss function, and the ‘Adam’ optimizer was used for optimization. The models were trained with 100 epochs and a batch size of 128. The proposed ensemble LSTM-GRNN is a combination of LSTM, GRU, and RNN, which were stacked to achieve significant performance.

### 3.6. Architecture of Proposed LSTM-GRNN

**Figure 2:**
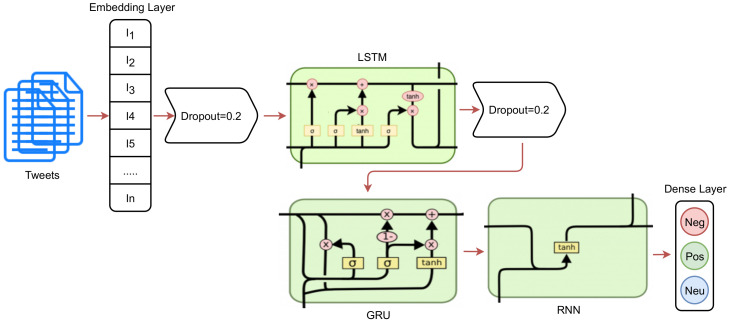
Flow diagram of proposed LSTM-GRNN architecture.

The proposed ensemble LSTM-GRNN makes use of the stacked LSTM, GRU, and RNN networks to obtain higher levels of accuracy for sentiment analysis. LSTM-GRNN consists of seven layers, as described in Table 9. It has one embedding layer, two dropout layers, one layer each for LSTM, GRU, and RNN, and a dense layer. The embedding layer is used with a vocabulary size of 5000 and output size of 300. The embedding layer is followed by a dropout layer, as shown in Figure 2. The dropout rate for this layer is 0.2 and the dropout is used to help reduce the complexity in the model and the probability of the model over-fitting. The LSTM layer is on the top of the stack with 100 LSTM units. The GRU layer follows the LSTM layer with 100 units. The RNN layer is at the end of the stack with 32 units, followed by a dropout rate of 0.2. In the end, a dense layer with 3 neurons and a softmax activation function was used to get the desired target classes. The LSTM-GRNN was fitted with 100 epochs and compiled using a categorical_crossentropy loss function and ’Adam’ optimizer.

### 3.7. Lexicon-Based Approach for Sentiment Analysis

**Figure 3:**
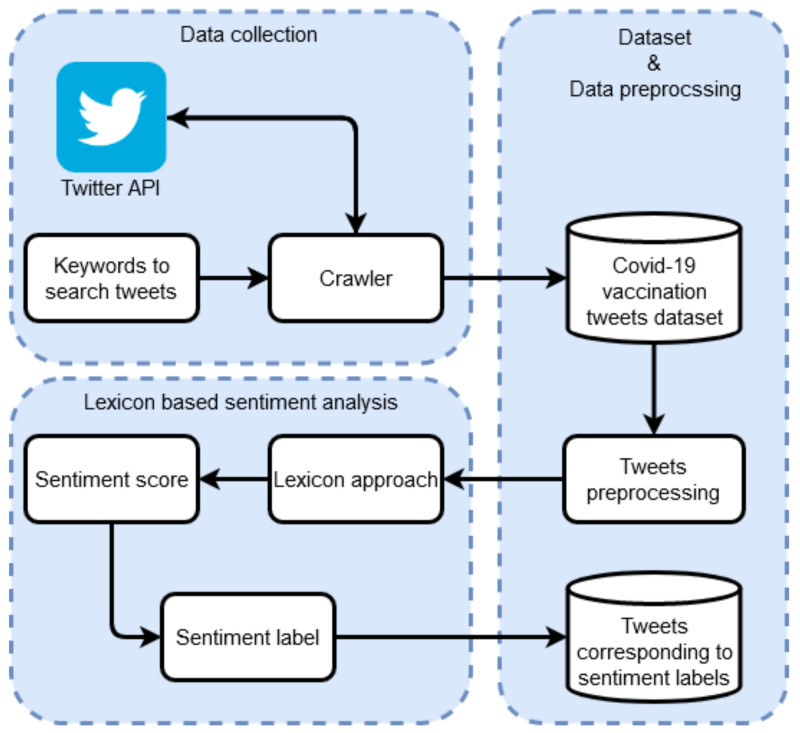
Lexicon-based approach for sentiment analysis.

Preprocessing techniques make the dataset clean, which can produce better results. After preprocessing, the dataset was analyzed to find the sentiments using three lexicon-based approaches. These lexicon-based approaches provide three sentiments as an output against each tweet. Three lexicon-based approaches, TextBlob, AFINN, and VADER, were used in this study. These approaches give polarity scores as their output to determine the sentiment. Moreover, a detailed analysis was performed on all the country-wise tweets to find people’s perceptions and concerns regarding the COVID-19 vaccination. Figure 3 illustrates the architecture of the lexicon approaches along with the steps and their sequence performed in this study.

### 3.8. Proposed Methodology for Sentiment Analysis

**Figure 4:**
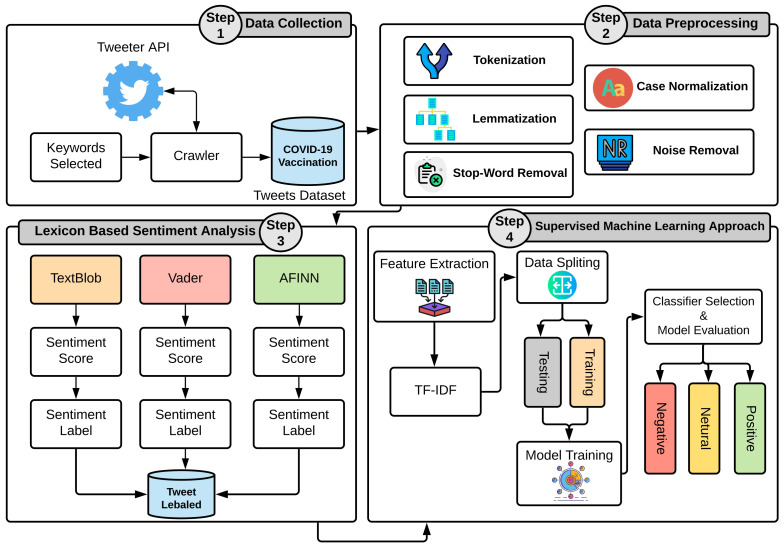
The architecture of the proposed methodology used for sentiment analysis.

After the lexicon-based approaches are used to determine the sentiment along with tweet labeling, the labeled tweets dataset was used for the training of the machine learning models. The trained models were used to classify the sentiments as positive, negative, or neutral. TF-IDF features were extracted from the labeled dataset, followed by the dataset split in 80 to 20 ratios for training and testing, respectively. An evaluation of the appropriate combinations of lexicon-based sentiment analysis approaches was carried out to analyze the performance of both lexicon and machine learning methods. For instance, the experiments were performed using all three TextBlob, AFINN, and VADER sentiments as target classes with the selected machine learning models to analyze the high-performing lexicon method. Similarly, the performance of the selected machine learning models RF, LR, and DT was carried out in terms of accuracy, precision, recall, and F1 score. Figure 4 shows the architecture of the proposed methodology used for sentiment analysis.

## 4. Results and Discussions

Experiments were performed using the Intel Core i7 7th generation machine with 8 GB RAM and the Windows 10 operating system. Python language was used to implement the script on Jupiter notebook. Machine learning models were implemented using the Scikit-learn library, while TensorFlow was used for deep learning models.

For sentiment analysis, the words, and sentences can be selected and analyzed to determine the sentiments regarding the selected topic. Sentiments can be determined as positive, negative, or neutral. For this purpose, this study relies on two techniques, namely, NLP-based lexicon methods and machine learning classification models, to determine the sentiments regarding the ongoing vaccination-related sentiments around the globe. Three NLP lexicon-based approaches were deployed, including the TextBlob, AFINN, and VADER, along with three machine learning models, including RF, LR, and DT. The following discussions aim at presenting and analyzing the performance of lexicon and machine learning methods for sentiment analysis.

**Figure 5:**
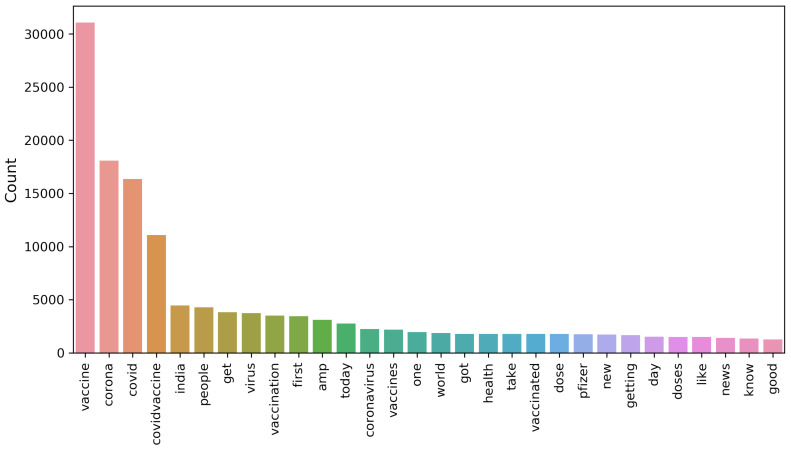
Chart for uni-gram terms from the collected dataset.

**Figure 6:**
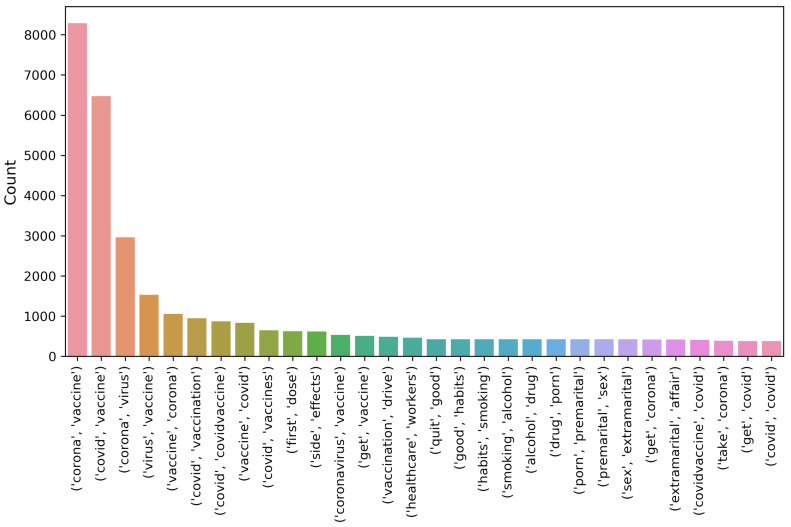
Chart for bi-gram terms from the collected dataset.

**Figure 7:**
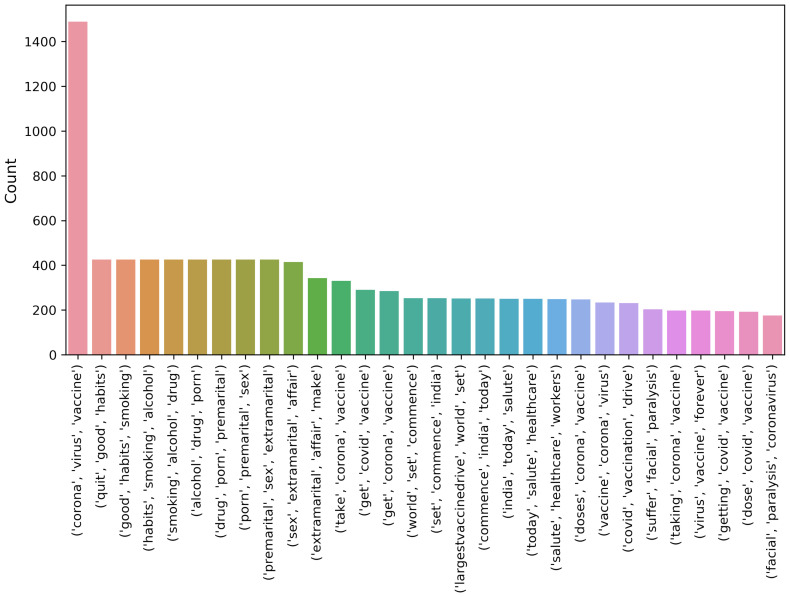
Chart for tri-gram terms from the collected dataset.

Figure 5, Figure 6 and Figure 7 show the uni-gram, bi-gram, and tri-gram distributions of the dataset of the COVID-19 vaccination. The uni-gram and bi-gram graphs show that the most commonly used words were ‘covid’, ‘vaccine’ and ‘covid’, while the tri-gram shows that the highly discussed topics were the COVID-19 vaccination campaign, COVID-19 vaccination for health workers, vaccination side-effects, receiving the first dose, and so forth.

**Figure 8:**
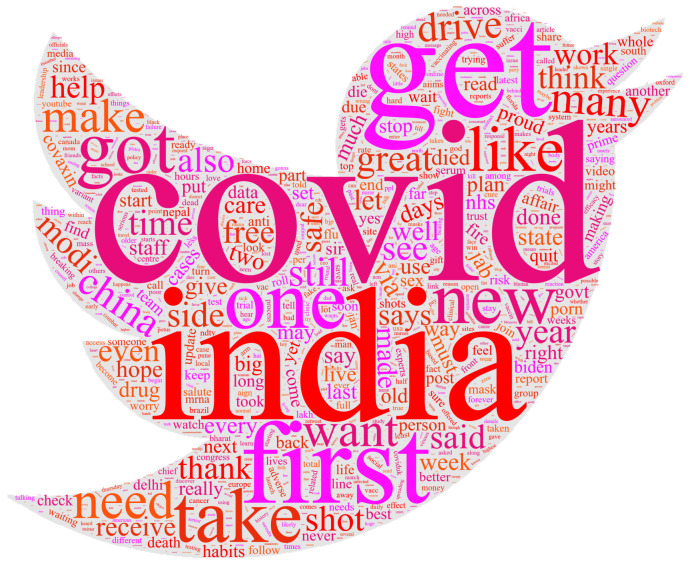
Wordcloud for the tweets dataset showing the most used words.

Figure 8 shows the word cloud of the dataset containing the perceptions and opinions of the people around the globe regarding the ongoing COVID-19 vaccination. Similar to the uni-gram, bi-gram, and tri-gram terms, the world cloud illustrates that ‘COVID’, ‘taking vaccination’, and ‘vaccination drive’, and so forth are the most commonly used words in the tweets.

### 4.1. POS Tags of Dataset

Table 10 shows the POS-tagged focused words with the corresponding count of words in the collected dataset. It contains different words along with their corresponding POS tags. For instance, nouns (NN) contain a subset of nouns used in the text of the tweets along with the word count. Similarly, adjective (JJ) represents the adjective words found in the dataset, and their total occurrences are given in the corresponding columns.

### 4.2. Sentiment Analysis Using TextBlob

**Figure 9:**
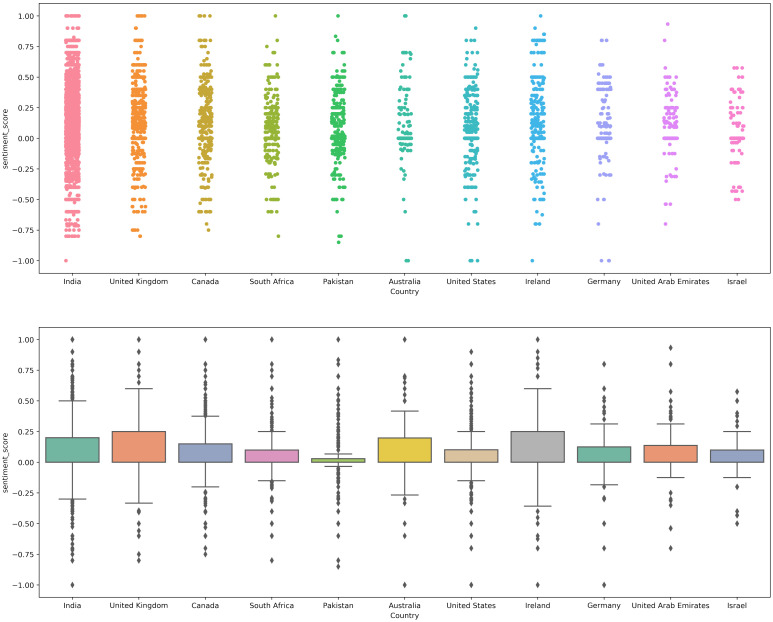
TextBlob sentiment score for tweets from different countries.

Experiments were carried out for each lexicon method separately. Figure 9 shows the sentiment polarity score using the TextBlob method. It shows that a higher number of tweets has a positive polarity score. Tweets for each country in specific and all tweets, in general, have a positive sentiment score of 0 to 0.3, indicating that although tweets are determined as positive, their average polarity score is low. We can say that the tweets are positive with less intensity because a higher number of positive tweets have a polarity score between 0.1 to 0.5 polarity score.

Table 11 shows the results of country-wise sentiment analysis along with the overall sum of all the countries using the TextBlob lexicon method. Tweet sentiments were categorized into positive, negative, and neutral. Each column shows the percentage of three sentiment categories regarding each country. Results indicate that the majority of the tweets belong to the neutral class, followed by the positive tweets, while the negative tweets are the lowest, considering the tweets from all the countries combined. The ratio of neutral, positive, and negative tweets is 48.81%, 38.33%, and 12.86%, respectively.

### 4.3. Sentiment Analysis Using VADER

**Figure 10:**
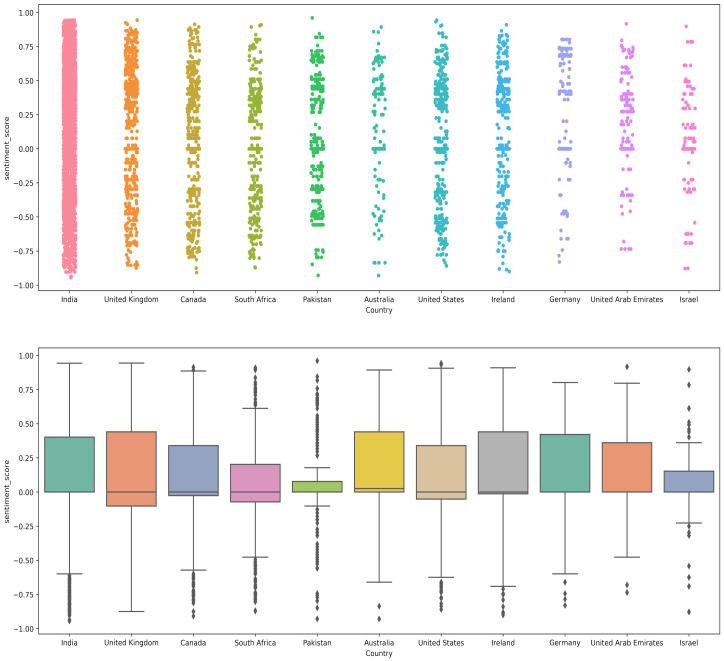
VADER sentiment scores for each country.

Figure 10 shows the polarity score given by the VADER approach. The displayed results indicate that VADER-assigned negative polarity scores are higher as compared to the TextBlob. TextBlob gives 12% negative tweets, while VADER assigns a negative polarity score to 22% indicating 10% higher negative tweets than the TextBlob.

**Figure 11:**
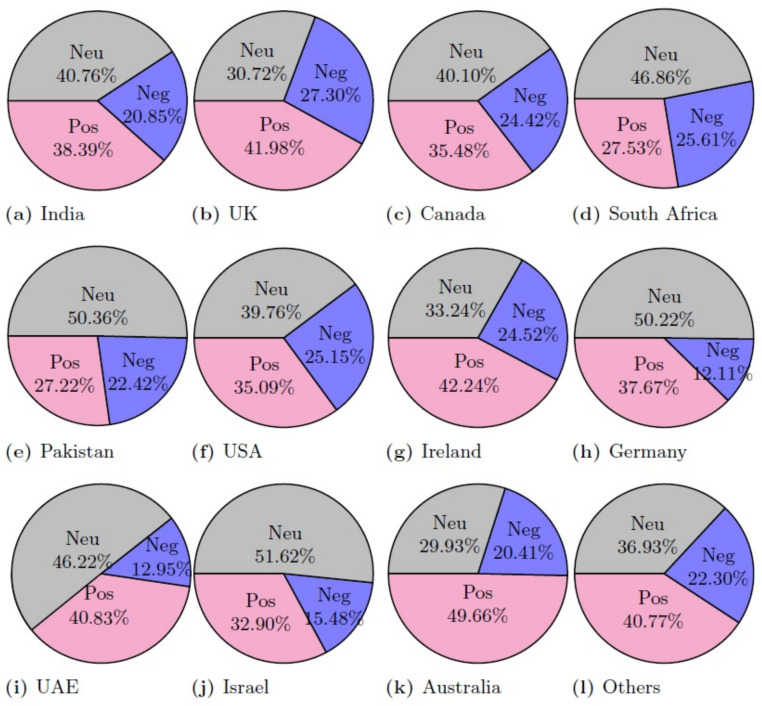
Percentage of the sentiment analysis for VADER.

Figure 11 and Table 12 shows the results of country-wise sentiment analysis along with the overall sum of all the countries using the VADER lexicon method. The ratio of positive, negative, and neutral tweets was changed, as compared to TextBlob. Although the change in the ratio of positive tweets is small, there is a substantial change in the ratio of neutral and negative tweets. For example, the ratio of neutral tweets was changed from 48.81% to 37.74% for VADER, while negative tweets were raised to 22.31% from 12.86%. It indicates that a large number of tweets with neutral sentiments from TextBlob was determined as negative when VADER was used.

### 4.4. Sentiment Analysis Using AFINN

**Figure 12:**
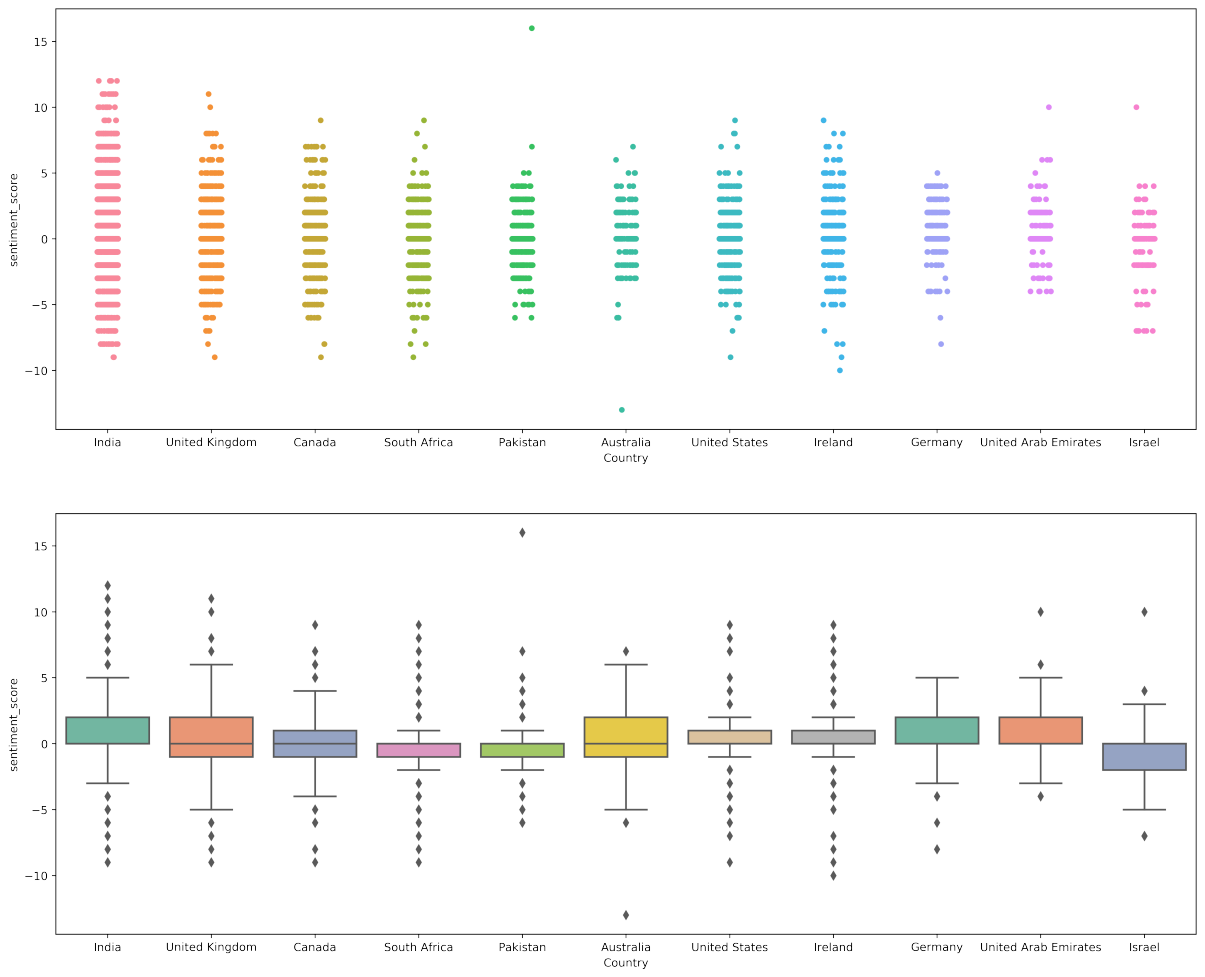
AFINN sentiment score for different countries.

Figure 12 shows the sentiment analysis results using the AFINN method on the collected dataset. Results indicate that similar to VADER, AFINN assigns a negative polarity score as compared to the TextBlob. Both country-wise tweets and collective tweets fall in the range of a 0 to −2 polarity score. Tweets with more negative sentiments were from countries like Israel, Germany, Australia, and Pakistan.

**Figure 13:**
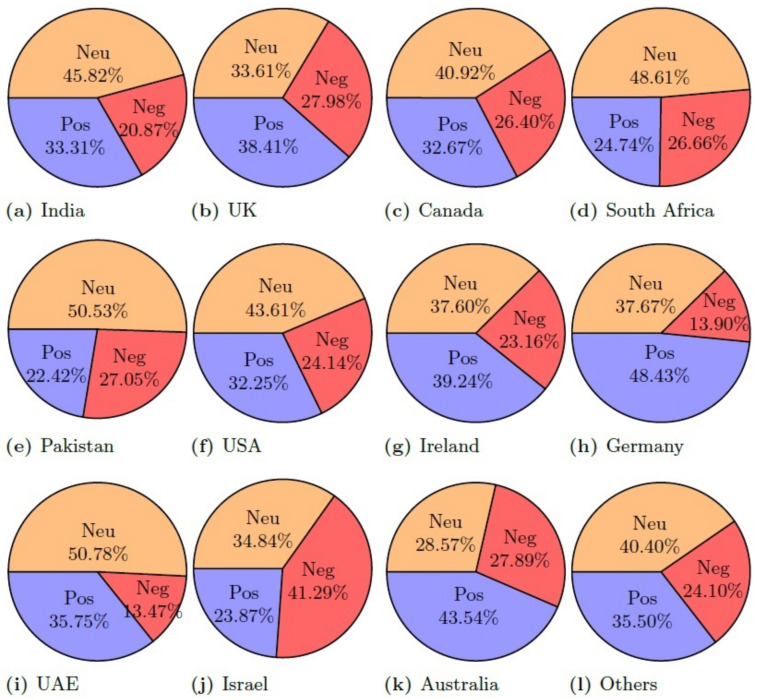
Percentage of the sentiment analysis for AFINN.

Figure 13 and Table 13 shows the results of country-wise sentiment analysis along with the overall sum of positive, negative, and neutral tweets using the AFINN lexicon method. Results indicate that the ratio of neutral tweets is similar to that of TextBlob, however, the ratio of negative tweets is higher than both TextBlob and VADER with 23.77% negative tweets. The positive and neutral tweets, on the other hand, are 35% and 41.21%, respectively.

**Figure 14:**
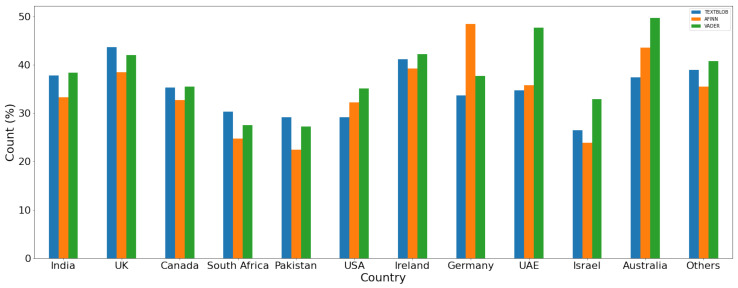
Positive sentiment (%) using each lexicon-based approach.

**Figure 15:**
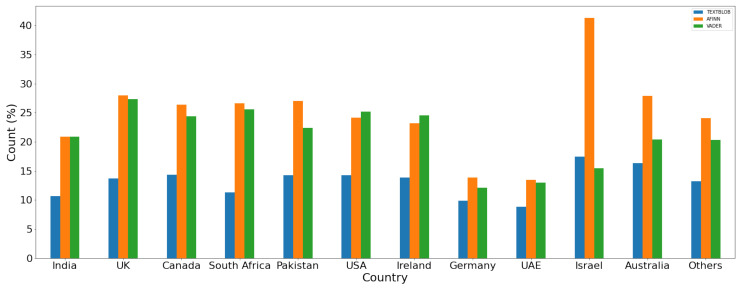
Negative sentiment (%) using each lexicon-based approach.

**Figure 16:**
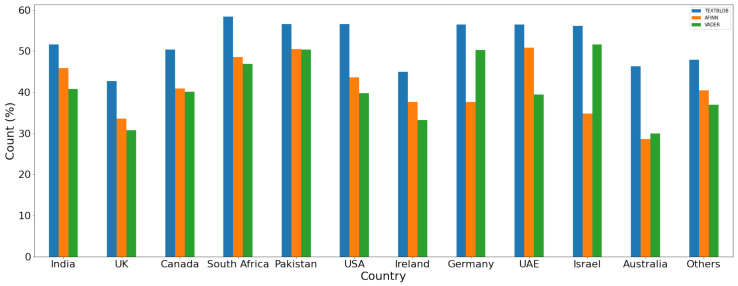
Neutral sentiment (%) using each lexicon-based approach.

For a comparison of the polarity of the sentiment for the given dataset, results are given in Figure 14, Figure 15 and Figure 16 for positive, negative, and neutral sentiments, respectively using the TextBlob, VADER, and AFFIN lexicon approach.

### 4.5. Sentiment Analysis Using Machine Learning Models

Besides using the lexicon-based methods, this study used several machine learning models for sentiment analysis on the annotated dataset related to COVID-19 vaccination tweets. Lexicon-based methods are utilized for calculating the sentiment score to determine the label of a tweet into positive, negative, or neutral using the polarity score by lexicon methods. The resulting dataset was used for the training and testing of the machine learning classifiers. All three models were trained and tested on the individual datasets annotated using TextBlob, AFINN, and VADER. The performance was evaluated using accuracy, precision, recall, and F1 score. The performance evaluation was done for each ML model using all three individual lexicon methods.

Table 14 shows the results for the machine learning models for accuracy and other performance evaluation metrics. The dataset annotated using the TextBlob was fed into the machine learning models for experiments. Results show that both RF and LR obtained the highest level of accuracy of 93% each in comparison to DT, which has 92% accuracy.

In addition to using the TextBlob annotated dataset, experiments were also performed with a VADER-labeled dataset. Table 15 shows the performance of the machine learning models when used with a VADER annotated dataset. Results demonstrate that both RF and LR have an equal performance of 90% accuracy when applied to the VADER sentiment analysis dataset. However, RF outperforms regarding precision, recall, as well as F1 score metrics among the three models. Additionally, the performance of the models was reduced substantially when the dataset was changed from TextBlob to annotated VADER. For example, the accuracy of both LR and RF was reduced to 90% from 93%, while DT experienced a substantial reduction to 82% from 92% when trained with a VADER-annotated dataset.

In the end, the AFINN-annotated dataset is used for sentiment analysis experiments using the selected machine learning models, and results are given in Table 16. Results indicate that RF obtained the highest performance in terms of accuracy, precision, recall, and F1 score. LR experienced a marginal reduction in its accuracy from 90% to 89% when the dataset was changed from VADER to AFINN. On the other hand, DT had a slight increase in the accuracy from 83% to 84% for the VADER and AFINN datasets, respectively.

Experimental results reveal that the models perform better when used with TextBlob-annotated data as compared to VADER and AFINN. Previous studies [50,51,52] show that models perform better when trained on TextBlob labeled data, and this study confirms the same.

### 4.6. Experimental Results of Deep Learning Models

Keeping in view the higher sentiment classification with the TextBlob annotated data, experiments for deep learning models are performed using the TextBlob dataset with accuracy, precision, recall, and F1 score as the evaluation parameters. Experimental results are provided in Table 17. Results suggest that the proposed LSTM-GRNN outperforms all other models in terms of all evaluation parameters. It achieved the highest accuracy of 95%, which is higher than both the machine and deep learning models used in this study. In addition to accuracy, precision, recall, and F1 scores were also higher than other models. GRU also performed better with 93% accuracy, followed by the LSTM and RNN each with 92% accuracy. The CNN model showed poor performance as compared to recurrent models because the CNN model requires a large feature set as compared to recurrent models, while CNN shows comparatively better performance when combined with LSTM.

### 4.7. Comparison with State-of-the-Art Studies

To represent the significant performance of the proposed LSTM-GRNN model in the context of other studies, a performance comparison was carried out with several other studies. For this purpose, the models proposed in selected studies were implemented using the collected dataset, and the results were compared with the results from this study. The study in [39] presented an ensemble model for sentiment classification, while the study in [39] has used LR-SGDC (stochastic gradient descent classifier) for US airline sentiments. Similarly, the study in [53] used an extra tree classifier (ETC) for the same task. In addition, the study in [54] used the CNN-LSTM model for sarcasm detection, and the study in [55] has performed sentiment analysis using the stacked Bi-LSTM model. For a fair comparison, these models were deployed using the COVID-19 vaccination tweets dataset that was collected in this study. Training and testing was performed using the TextBlob annotated dataset, and a performance comparison is given in Table 18. Results suggest that the proposed approach is significantly better than other studies in terms of accuracy. Despite using the ensemble models in other studies, the proposed LSTM-GRNN with a TextBlob-annotated dataset showed superior performance and obtained 95% accuracy for sentiments, which is higher than previous studies.

### 4.8. Time-Based Sentiment Analysis

**Figure 17:**
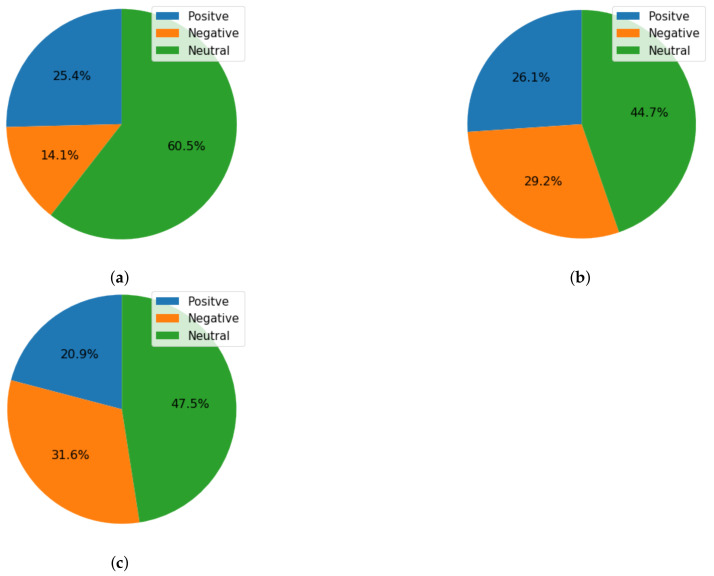
Ratio of sentiments for COVID-19 vaccination-related tweets for January 2020, (**a**) TextBlob sentiments, (**b**) VADER sentiments, and (**c**) AFINN sentiments.

Time-based sentiment analysis was also performed to analyze the change in trends of people regarding COVID-19 sentiments. A new set of tweets was collected for January 2022 and performed sentiment analysis using the Vader, TextBlob, and Afinn techniques. The ratio of positive, negative, and neutral sentiments based on each lexicon technique is shown in Figure 17a–c. Results suggest that the ratio of negative tweets increased for COVID-19 vaccinations as compared to 2021 tweets.

The ratio of positive, negative, and neutral sentiments using TextBlob was 38.33%, 12.86%, and 48.81%, respectively for 2021 tweets, which were changed to 25.40%, 14.10%, and 60.50%, respectively. A comparative analysis for each lexicon technique is given in Table 19.

## 5. Conclusions

Vaccination of the whole population at a fast pace is encouraged by the WHO to minimize the spread and fatality risks, and governments are utilizing all available resources to accelerate COVID-19 vaccinations. Despite recommendations to take the vaccine from government officials, medical experts, and social workers, people show concerns and reservations regarding the side effects and other medical complications that may arise when vaccinated. This study proposes a methodology to analyze the global perceptions and perspectives of people towards COVID-19 vaccinations using a worldwide Twitter dataset. Dataset analysis indicates that the majority of the tweets in the collected dataset belongs to the neutral and positive classes regarding the COVID-19 vaccination. The study relies on two techniques: the NLP lexicon-based method for annotating the sentiments, and machine and deep learning models for sentiment analysis. Experimental results using TextBlob, VADER, and AFINN show that machine learning models show good performance with a TextBlob-labeled dataset with a 93% accuracy score using DT and LR. For increasing the sentiment classification accuracy, LSTM-GRNN, the ensemble of LSTM, GRU, and RNN, is proposed. Results reveal that LSTM-GRNN performs significantly better than all the machine learning and deep learning models used in this study. Furthermore, a performance comparison with state-of-the-art models proves the model’s superiority for sentiment classification with a 95% accuracy score. The decision-making process towards an effective and successful vaccination drive may be guided by engagement with the target population by listening and responding to their concerns, expectations, and difficulties related to the vaccination. Time-based sentiment analysis shows that the ratio of negative sentiments for 2022 was increased as compared to 2021.

### 5.1. Findings of Research

The key findings of this research are as follows.

- The ratio of positive sentiments is high as compared to the ratio of negative sentiments in tweets related to COVID-19 vaccinations.
- The ratio of sentiments for positive, negative, and neutral sentiments may vary, yet, on average, the number of neutral sentiments is higher than negative and positive sentiments.
- Time-based analysis of tweets related to COVID-19 vaccination indicates a negative trend, that is, the ratio of negative sentiments slightly increased over time.
- Tree-based machine learning models proved perform better than other models. Ensemble models can be a good choice for obtaining higher levels of classification accuracy when dealing with tweets’ textual data.
- Regarding the performance of lexicon-based approaches, the use of TextBlob for annotation leads to higher levels of performance.

### 5.2. Limitations and Future Work

This study collected the data from Twitter for conducting sentiment analysis about the COVID-19 vaccination. The collected data were processed to remove noise and redundant information; however, the aspect of fake news was not handled. Since the probability of fake tweets cannot be ignored, it implies that finding and removing the fake tweets may affect and change the performance of classifiers. Similarly, the analysis aims at perceptions and conceptions of people regarding the vaccination, and no specific vaccine was targeted that would otherwise provide a better picture of people’s sentiments regarding specific vaccines. We intend to cover these aspects in the future. We are also looking forward to developing a system that is capable of performing real-time sentiment analysis and determining social trends for effective decision-making.

## Figures and Tables

**Table 1:** Sample text from tweets dataset.

User Name	Location	Tweets
lunini	Washington DC	As expected WHO celebrates return of # USA to the organization during the surge of the covid # pandemic # COVID19$\hat{a}$€| https://t.co/TbVcBF3Nxr (accessed on: 20 May 2021)
danschoenmn	St. Paul Park	We$\hat{a}$€${}^{TM}$re learning there was no federal plan to get the vaccine to our citizens. NONE! Imagine knowing something is har$\hat{a}$€| https://t.co/XU9ADtpNlV (accessed on: 20 May 2021)
RichardILevine	Hawaii, USA	@drdavidsamadi And THAT is how you end a pandemic. And credit will go to the # vaccine. where did we see this cleri$\hat{a}$€| https://t.co/X6OnOhbiMs (accessed on: 20 May 2021)
FrancicoCabral	Lisboa	There$\hat{a}$€${}^{TM}$s only one way forward: every person on earth will either get the virus or the vaccine. # COVID19 # vaccine

**Table 2:** Data after removal of username, hashtags, and hyperlinks.

Tweets before Removal	Tweets after Removal
Many thyroid and autoimmune patients wonder whether they should get the COVID vaccine. Thyroid Expert Mary S$\hat{a}$€| https://t.co/8OHcyR5kQ7 (accessed on: 20 May 2021)	Many thyroid and autoimmune patients are wondering whether they should get the COVID vaccine. Thyroid Expert Mary S$\hat{a}$€|
As expected @WHO celebrates return of # USA to the organization during the surge of the covid #pandemic #COVID19$\hat{a}$€| https://t.co/TbVcBF3Nxr (accessed on: 20 May 2021)	As expected celebrates return of to the organization during the surge of the covid |

**Table 3:** Data after removal of numbers, punctuations, and stopwords.

Tweets before Removal	Tweets after Removal
Many thyroid and autoimmune patients are wondering whether they should get the COVID vaccine. Thyroid Expert Mary S$\hat{a}$€|	Many thyroid autoimmune patients wondering whether get COVID vaccine. Thyroid Expert Mary
As expected, celebrates return of to the organization during the surge of the covid |	expected celebrates return organization surge covid

**Table 4:** Data after Lower case conversion, stemming, and lemmatization.

Tweets before Removal	Tweets after Removal
Many thyroid autoimmune patients are wondering whether to get the COVID vaccine. Thyroid Expert Mary	many thyroid autoimmune patient wonder whether get covid vaccine thyroid expert mary
expected celebrates return organization surge covid	expect celebrate return organization surge covid

**Table 5:** Data before and after preprocessing.

Before Preprocessing	After Preprocessing
Many thyroid and autoimmune patients are wondering whether they should get the COVID-19 vaccine. Thyroid Expert Mary S$\hat{a}$€| https://t.co/8OHcyR5kQ7 (accessed on: 20 May 2021)	many thyroid autoimmune patient wonder whether get covid vaccine thyroid expert mary
As expected, @WHO celebrated the return of #USA to the organization during the surge of the covid #pandemic #COVID19$\hat{a}$€| https://t.co/TbVcBF3Nxr (accessed on: 20 May 2021)	expect celebrate return organization surge covid

**Table 6:** TextBlob sentiment score range.

Sentiment	Score
Negative	Polarity score < 0
Neutral	Polarity score = 0
Positive	Polarity score > 0

**Table 7:** VADER sentiment score range.

Sentiment	Score
Negative	compound score <= −0.05
Neutral	compound score > −0.05 to compound score < 0.05
Positive	compound score >= 0.05

**Table 8:** AFINN sentiment score range.

Sentiment	Score
Negative	Polarity score < 0
Neutral	Polarity score = 0
Positive	Polarity score > 0

**Table 9:** Architecture of deep learning models.

LSTM	CNN	RNN
Embedding (5000, 200) Dropout (0.2) LSTM (100) Dropout (0.2) Dense (3, activation = ‘softmax’)	Embedding (5000, 200) Dropout (0.2) Conv1D (128, 4, activation = ‘relu’) MaxPooling1D (pool_size = 4) Flatten () Dense (32) Dense (2, activation = ‘softmax’)	Embedding (5000, 200) Dropout (0.2) SimpleRNN (32) Dense (3, activation = ‘softmax’)
**GRU**	**CNN-LSTM**	**LSTM-GRNN**
Embedding (5000, 200) Dropout (0.2) GRU (100) Dropout (0.2) Dense (3, activation = ‘softmax’)	Embedding (5000, 200) Dropout (0.2) Conv1D (128, 4, activation = ‘relu’) MaxPooling1D (pool_size = 4) LSTM (128) Dense (32) Dense (3, activation = ‘softmax’)	Embedding (5000, 200) Dropout (0.2) LSTM (100) Dropout (0.2) GRU (100) SimpleRNN (32) Dense (3, activation = ‘softmax’)
loss = ‘categorical_crossentropy’, optimizer = ‘adam’, epochs = 100		

**Table 10:** POS tagging.

NN	Count	JJ	Count	Entity Name	Entity Type	Count
Vaccine	30,209	Corona	4483	India	GPE	3033
Virus	3540	Good	1262	Today	DATE	1787
India	2686	Dose	1080	First	ORDINAL	1557
World	1879	Many	1052	China	GPE	635
Health	1791	Great	894	Million	CARDINAL	503
Pfizer	1587	Free	789	Pakistan	GPE	473
Country	1525	Safe	739	Pfizer	ORG	428
Worker	1405	Pandemic	665	Healthcare	ORG	413
News	1403	Medical	608	Norway	GPE	404
Government	991	Premarital	425	Chinese	NORP	288

**Table 11:** TextBlob sentiment statistics for each country and worldwide.

Country	Positive	Negative	Neutral
All Countries	38.33	12.86	48.81
India	37.74	10.66	51.60
United Kingdom	43.62	13.72	42.66
Canada	35.31	14.36	50.33
South Africa	30.31	11.32	58.36
Pakistan	29.18	14.23	56.58
United State	29.18	14.23	56.58
Ireland	41.14	13.90	44.96
Germany	33.63	9.87	56.50
UAE	34.72	8.81	56.48
Israel	26.45	17.42	56.13
Australia	37.41	16.33	46.253
Other Countries	38.92	13.25	47.83

**Table 12:** VADER sentiment statistics for each country and worldwide.

Country	Positive (%)	Negative (%)	Neutral (%)
All Countries	39.95	22.31	37.74
India	38.39	20.85	40.75
United Kingdom	41.97	27.30	30.73
Canada	35.48	24.42	40.10
South Africa	27.53	25.61	46.86
Pakistan	27.22	22.42	50.36
United State	35.09	25.15	39.76
Ireland	42.23	24.52	33.24
Germany	37.67	12.11	50.22
UAE	47.67	12.95	39.39
Israel	32.90	15.48	51.61
Australia	49.66	20.41	29.93
Others Countries	40.77	22.30	36.93

**Table 13:** AFFIN sentiment statistics for each country and worldwide.

Country	Positive (%)	Negative (%)	Neutral (%)
Total	35.01	23.78	41.22
India	33.31	20.87	45.83
United Kingdom	38.41	27.98	33.61
Canada	32.67	26.40	40.92
South Africa	24.74	26.65	48.61
Pakistan	22.42	27.05	50.53
United State	32.25	24.14	43.61
Ireland	39.24	23.16	37.60
Germany	48.43	13.90	37.67
UAE	35.75	13.47	50.78
Israel	23.87	41.29	34.84
Australia	43.54	27.89	28.57
Other Country	35.50	24.10	40.40

**Table 14:** Performance results for machine learning models using TextBlob sentiments.

Model	Accuracy (%)	Precision (%)	Recall (%)	F1 Score (%)
DT	92	93	87	90
RF	93	96	92	94
LR	93	94	87	89

**Table 15:** Machine learning model performances on a VADER sentiment.

Model	Accuracy (%)	Precision (%)	Recall (%)	F1 Score (%)
DT	83	86	81	82
RF	90	92	89	90
LR	90	91	88	89

**Table 16:** Machine learning model performance on the AFINN sentiment.

Model	Accuracy (%)	Precision (%)	Recall (%)	F1 Score (%)
DT	84	87	81	83
RF	90	92	89	90
LR	89	90	88	89

**Table 17:** Deep learning model performance on TextBlob sentiment.

Model	Accuracy (%)	Precision (%)	Recall (%)	F1 Score (%)
LSTM	92	90	90	90
GRU	93	93	93	92
RNN	92	92	92	92
CNN	87	87	87	87
CNN-LSTM	88	88	88	88
LSTM-GRNN	95	95	95	95

**Table 18:** Performance comparison with previous studies.

Ref.	Year	Model	Accuracy (%)
[39]	2019	LR-SGDC	90
[53]	2021	ET + FU	91
[54]	2021	CNN-LSTM	88
[55]	2021	Stacked Bi-LSTM	93
This study	2021	LSTM-GRNN	95

**Table 19:** Comparison of change in the sentiments over time.

Year	Ratio of Sentiments		
	Positive	Negative	Neutral
TextBlob			
2021	38.33	12.86	48.81
2022	25.40	14.10	60.50
VADER			
2021	39.95	22.31	37.74
2022	26.10	29.20	44.70
AFINN			
2021	35.01	23.78	41.22
2022	20.90	31.60	47.50

## Data Availability

Not applicable.
